# Development of TaqMan-Based Quantitative PCR for Sensitive and Selective Detection of Toxigenic *Clostridium difficile* in Human Stools

**DOI:** 10.1371/journal.pone.0111684

**Published:** 2014-10-31

**Authors:** Hiroyuki Kubota, Takafumi Sakai, Agata Gawad, Hiroshi Makino, Takuya Akiyama, Eiji Ishikawa, Kenji Oishi

**Affiliations:** 1 Yakult Honsha European Research Center for Microbiology ESV, Gent-Zwijnaarde, Belgium; 2 Yakult Central Institute, Tokyo, Japan; Institute Pasteur, France

## Abstract

**Background:**

*Clostridium difficile* is the main cause of nosocomial diarrhea, but is also found in asymptomatic subjects that are potentially involved in transmission of *C. difficile* infection. A sensitive and accurate detection method of *C. difficile*, especially toxigenic strains is indispensable for the epidemiological investigation.

**Methods:**

TaqMan-based quantitative-PCR (qPCR) method for targeting 16S rRNA, *tcdB*, and *tcdA* genes of *C. difficile* was developed. The detection limit and accuracy of qPCR were evaluated by analyzing stool samples spiked with known amounts of *C. difficile*. A total of 235 stool specimens collected from 82 elderly nursing home residents were examined by qPCR, and the validity was evaluated by comparing the detection result with that by *C. difficile* selective culture (CDSC).

**Results:**

The analysis of *C. difficile*-spiked stools confirmed that qPCR quantified whole *C. difficile* (TcdA^+^TcdB^+^, TcdA^−^TcdB^+^, and TcdA^−^TcdB^−^ types), TcdB-producing strains (TcdA^+^TcdB^+^ and TcdA^−^TcdB^+^ types), and TcdA-producing strains (TcdA^+^TcdB^+^ type), respectively, with a lower detection limit of 10^3^ cells/g of stool. Of the 235 specimens examined, 12 specimens (5.1%) were *C. difficile*-positive by qPCR: TcdA^+^TcdB^+^ strain in six specimens and TcdA^−^TcdB^−^ strain in the other six. CDSC detected *C. difficile* in 9 of the 12 specimens, and toxigenic types of the isolates from the 9 specimens were consistent with those identified by qPCR, supporting the validity of our qPCR method. Moreover, the qPCR examination revealed that the carriage rate of whole *C. difficile* and that of toxigenic strains in the 82 subjects over a 6-month period ranged from 2.4 to 6.8% and 1.2 to 3.8%, respectively. An average qPCR count of *C. difficile* detected was 10^4.5^ cells/g of stool, suggesting that *C. difficile* constituted a very small fraction of intestinal microbiota.

**Conclusion:**

Our qPCR method should be an effective tool for both clinical diagnosis and epidemiological investigation of *C. difficile*.

## Introduction

*Clostridium difficile* is the most common cause of healthcare-associated infectious diarrhea [1]. This bacterium can be categorized into three types on the basis of its production of two major toxins, TcdA and TcdB, namely i) TcdA-positive, TcdB-positive (A^+^B^+^) type; ii) TcdA-negative, TcdB-positive (A^−^B^+^) type; and iii) TcdA-negative, TcdB-negative (A^−^B^−^) type. Toxigenic strains, A^+^B^+^ and A^−^B^+^ types, are responsible for *C. difficile* infection (CDI). The elderly are particularly at increased risk of CDI possibly because of age-related changes in intestinal microbiota, weakened immune systems, and the presence of underlying diseases [2]. On the other hand, several surveys have reported that toxigenic *C. difficile* was detected in stools of asymptomatic elderly people [3]–[5]; such asymptomatic carriers may play a role in the transmission of CDI [5]. Selective detection of toxigenic *C. difficile* strains is therefore important, not only for clinical diagnosis but also for epidemiological investigations of *C. difficile* for infection control. Moreover, the quantification method of toxigenic *C. difficile* is useful in basic research such as studies to evaluate correlation of *C. difficile* count in the intestine and severity of the CDI symptom or to find an association between *C. difficile* colonization and the presence or absence of other intestinal bacteria.

*C. difficile*, including toxigenic strains in stool specimens, is traditionally detected by the culture method using selective medium. The *C. difficile* selective culture (CDSC) is a valuable method with advantages, such as high detection sensitivity and availability of isolates for characterization. However, the culture method is generally labor-intensive and time-consuming. In addition, since the culture method identifies predominant strains of *C. difficile* in stools, toxigenic strains that coexist with a larger population of non-toxigenic strains can be overlooked, which can fall into a false-negative result of toxigenic *C. difficile* detection. PCR-based methods have solved these shortcomings and have been widely used to detect toxigenic *C. difficile* in stools [6]–[9]. Several kinds of commercially available PCR kits are reported to be useful and reliable [10], [11]. However, their quantitative application remains insufficient. A recent study indicated the possibility of quantification by showing that the threshold cycle (*C_T_*) values of a commercial PCR targeting the *tcdB* gene were inversely correlated with *C. difficile* culture counts [12].

The purpose of this study was to develop a sensitive quantification method of toxigenic *C. difficile* and to evaluate its validity. We developed a new TaqMan real-time quantitative PCR (qPCR) method using sets of primers and a double-labeled fluorescent probe targeting the 16S rRNA, *tcdA*, and *tcdB* genes, and analyzed *C. difficile* in stools collected from elderly residents of nursing homes by means of qPCR.

## Materials and Methods

### Reference strains and culture conditions

All of the *C. difficile* strains (DSM 1296^T^, ATCC 43255, ATCC 43596, ATCC 43598, ATCC 700057, CCUG 20309, CCUG 37780, CCUG 37785, NTCT 13307, NTCT 13366) and the other organisms belonging to the *Clostridium* genus that we used were cultured anaerobically in modified Gifu anaerobic medium broth (Nissui Pharmaceutical Co., Ltd., Tokyo, Japan) supplemented with 1% glucose (1% Glc-mGAM) at 37°C. Total bacterial cell counts of fresh cultures were determined by using the 4′, 6-diamidino-2-phenylindole (DAPI) staining method in accordance with the method of Jansen *et al*. [13]. On the basis of the DAPI counts, each fresh culture was diluted to obtain 1 mL of bacterial suspension containing 10^9^ cells. Twenty microliters of suspension containing 2×10^7^ cells was stored at –80°C until use for DNA extraction.

### Development of primers and probes

The primers and probes listed in Table 1 were newly developed for this study. 16S rRNA gene-targeted primers (CD16SrRNA-F/R) and a probe (CD16SrRNA-P) for the detection of whole *C. difficile* (both toxigenic and non-toxigenic strains) were designed as follows. The sequences of *C. difficil*e strains and other clostridial species were obtained from Ribosomal Database Project (http://rdp.cme.msu.edu/). Multiple alignments of these genes with the Clustal X program (http://www.clustal.org/clustal2/) were used to identify highly conserved regions as targets of the *C. difficile* specific primers and probe. For the detection of TcdA-producing strains (A^+^B^+^) and TcdB-producing strains (A^+^B^+^ and A^−^B^+^), respective primers-probe sets targeting *tcdA* (tcdA-F/R/P) and *tcdB* (tcdB-F/R/P) were developed by using a procedure similar to that with CD16SrRNA-F/R/P. The sequences of *tcdA* and *tcdB* of *C. difficile* and other Large Clostridial Toxin genes as references obtained from GenBank were used for multiple alignments and identification of target sites. The accession numbers of gene sequences used for the design of the primers and probes were listed in Table S1.

**Table 1:** Oligonucleotide sequences used in this study.

Target gene	Oligonucleotide	Sequence (5′–3′)	Position	Amplicon size (bp)
16s rRNA	CD16SrRNA-F	GCAAGTTGAGCGATTTACTTCGGT	59–82^a^	155
	CD16SrRNA-P	FAM-TGCCTCTCAAATATATTATCCCGTATTAG-TAMRA	156–184^a^	
	CD16SrRNA-R	GTACTGGCTCACCTTTGATATTYAAGAG	186–213^a^	
*tcdA*	tcdA-F	CAGTCGGATTGCAAGTAATTGACAAT	6051–6076^b^	102
	tcdA-P	FAM-TTGAGATGATAGCAGTGTCAGGATTG-TAMRA	6124–6152^b^	
	tcdA-R	AGTAGTATCTACTACCATTAACAGTCTGC	6091–6116^b^	
*tcdB*	tcdB-F	TACAAACAGGTGTATTTAGTACAGAAGATGGA	6079–6110^c^	240
	tcdB-P	FAM-TTTKCCAGTAAAATCAATTGCTTC-TAMRA	6159–6182^c^	
	tcdB-R	CACCTATTTGATTTAGMCCTTTAAAAGC	6291–6318^c^	

a GenBank accession number NR074454.

b GenBank accession number M30307.

c GenBank accession number X53138.

### Stool specimens

Stool specimens were collected from 82 elderly residents from four nursing homes in France (11 males and 71 females; ages 66 to 94 years [average ± standard deviation, 84±6.2 years]). Stools were collected once every 3 months, three times in total (S_1_, S_2_, and S_3_) from each subject. Since no subjects had abdominal symptoms at these samplings, no diarrheal stools or CDI-suspected stools were included. The subject ID was composed of five digits: the first two digits specified the site number (from 01 to 04) and the latter three digits specified the subject number (from 001).

### Ethics statement

This study was conducted in compliance with the Declaration of Helsinki, good clinical practice (ICH topic E6; CPMP/ICH/135/95), and applicable regulatory requirements (French Public Health Code). The Independent Ethics Committee in Caen in France, COMITE DE PROTECTION DES PERSONNES NORD OUEST III, approved this study. Written informed consent forms were obtained from the subjects or their legal representatives.

### Preparation of stool specimens for each analysis

Stool specimens within 24 h after defecation were collected and immediately examined for *C. difficile* toxins by enzyme immunoassay (EIA) at study sites. A portion of each stool was concurrently collected into an empty tube, and stored at –20°C until transportation. The stool samples were transported in a frozen state from the sites to our laboratory, and stored at –20°C until use for the following pretreatment for qPCR and CDSC.

After being thawed, each stool was weighed and suspended in 9 volumes of Dulbecco’s PBS (–) (Nissui Pharmaceutical) to make a 10% (w/v) stool homogenate (100 mg stool/mL). One hundred microliters of stool homogenate was used immediately for CDSC. Two milliliters of the 10% stool homogenate was centrifuged at 16,000*×g* for 5 min, and the supernatant was discarded. The stool pellets (200 mg) were stored at –80°C until use for DNA extraction.

### DNA extraction

DNA was extracted from pure cultured bacteria and stool pellets by using a FastDNA SPIN Kit for Feces (MP Biomedicals, Illkirch, France) in accordance with the manufacturer’s instructions, with the exception that the first wash step with glass beads, sodium phosphate buffer, and PLS solution provided in the kit was skipped only in the extraction from pure culture bacteria. This is because the wash resulted in reduction in yield probably due to a difficulty of precipitation of bacterial cells with only glass beads in the buffer. DNA extracted from the pure cultured bacteria (2×10^7^ cells) or 200 mg of stool pellets was finally dissolved in 100 µL of provided buffer.

### qPCR

qPCR was performed in 384-well optical plates on an ABI PRISM 7900HT Sequence Detection System (Life Technologies, Foster City, CA). Ampdirect Plus (Shimadzu, Kyoto, Japan), a commercial PCR buffer, was used to neutralize inhibitory factors in stool DNA templates. Each reaction mixture of 20 µL was composed of 0.4 units of ExTaq DNA polymerase (TaKaRa Bio Inc., Shiga, Japan), 10 µL of 2×Ampdirect plus, 0.4 µL of Rox dye (Life Technologies), 0.2 µM of each specific primer, 0.2 µM of the fluorescent probe, and 5 µL of template DNA. The amplification program consisted of one cycle at 95°C for 30 s and then 50 cycles at 95°C for 5 s and 56°C for 50 s.

### Selectivity of qPCR using new primers-probe sets

The selectivity of qPCR using the newly designed primers-probe sets was determined. DNA fractions extracted from pure cultures of each strain shown in Table 2 at doses corresponding to 10^5^ cells per reaction were applied to qPCR with 16SrRNA-F/R/P, tcdA-F/R/P, and tcdB-F/R/P. *C_T_* values within ±3.3 of that obtained with the standard strain (*C. difficile* DSM 1296^T^) were considered positive (+), whereas *C_T_* values of more than 50 were considered negative (–) (Table 2). To evaluate the accuracy of quantitative detection among the target *C. difficile* strains, each analytical curve of the 10 *C. difficile* strains was generated by using the respective primers-probe sets (Table 3).

**Table 2:** Specific detection of target *C. difficile* strains by qPCR with newly developed oligonucleotide sets.

Taxon	Strain	Toxin production type^a^	Reactions with following oligonucleotide sets^b^ :		
			CD16SrRNA-F/R/P	tcdB-F/R/P	tcdA-F/R/P
*Clostridium difficile*	DSM 1296^T^	A^+^ B^+^	+	+	+
	ATCC 43255	A^+^ B^+^	+	+	+
	ATCC 43596	A^+^ B^+^	+	+	+
	NTCT 13307	A^+^ B^+^	+	+	+
	NTCT 13366	A^+^ B^+^	+	+	+
	ATCC 43598	A^−^ B^+^	+	+	−
	CCUG 20309	A^−^ B^+^	+	+	−
	ATCC 700057	A^−^ B^−^	+	−	−
	CCUG 37780	A^−^ B^−^	+	−	−
	CCUG 37785	A^−^ B^−^	+	−	−
*Clostridium bifermentans*	DSM 14991^T^	na	−	−	−
*Clostridium histolyticum*	DSM 2158^T^	na	−	−	−
*Clostridium innocuum*	DSM 1286^T^	na	−	−	−
*Clostridium novyi*	DSM 14992^T^	na	−	−	−
*Clostridium perfringens*	DSM 756^T^	na	−	−	−
*Clostridium ramosum*	DSM 1402^T^	na	−	−	−
*Clostridium septicum*	DSM 7534^T^	na	−	−	−
*Clostridium sordellii*	DSM 2141^T^	na	−	−	−
*Clostridium sphenoides*	DSM 632^T^	na	−	−	−
*Clostridium tertium*	DSM 2485^T^	na	−	−	−

a na, not applicable.

b The reactivity of qPCR for the target bacteria with each primers-probe set was investigated by using DNA extracts corresponding to 10^5^ cells per reaction from each pure culture of the listed strains. Reactivity was judged by using the criteria described in the Materials and Methods. In addition, negative PCR results were obtained for the following bacterial strains, representing the major intestinal bacteria: *Blautia productus* JCM 1471^T^, *Faecalibacterium prausnitzii* ATCC 27768^T^, *Bacteroides vulgatus* ATCC 8482^T^, *Bacteroides ovatus* JCM 5824^T^, *Fusobacterium varium* ATCC 8501^T^, *Collinsella aerofaciens* ATCC 25986^T^, *Prevotella melaninogenica* ATCC 25845^T^, *Veillonella parvula* GIFU 7884^T^, *Bifidobacterium longum* ATCC 15707^T^, *Bifidobacterium adolescentis* ATCC 15703^T^, *Bifidobacterium catenulatum* ATCC 27539^T^, *Lactobacillus gasseri* DSM 20243^T^.

**Table 3:** Comparison of qPCR analytical curves among *C. difficile* strains.

*C. difficile* strain	Toxin production type	CD16SrRNA-F/R/P		tcdB-F/R/P		tcdA-F/R/P	
		Analytical curve^a^	Δ*C_T_*value atx = 0^b^	Analytical curve^a^	Δ*C_T_* value at x = 0^b^	Analytical curve^a^	Δ*C_T_* value at x = 0^b^
DSM 1296^T^	A^+^ B^+^	y = −3.46×+40.0	–	y = −3.63×+44.4	–	y = −3.43×+42.9	–
ATCC 43255	A^+^ B^+^	y = −3.48×+39.9	−0.1	y = −3.58×+43.9	−0.5	y = −3.54×+43.0	0.1
ATCC 43596	A^+^ B^+^	y = −3.44×+39.1	−0.9	y = −3.63×+43.4	−1.0	y = −3.58×+42.6	−0.3
NTCT 13307	A^+^ B^+^	y = −3.42×+40.4	0.4	y = −3.64×+44.5	0.1	y = −3.57×+43.6	0.7
NTCT 13366	A^+^ B^+^	y = −3.46×+41.1	1.1	y = −3.67×+45.2	0.8	y = −3.56×+44.4	1.5
ATCC 43598	A^−^ B^+^	y = −3.47×+40.2	0.2	y = −3.58×+44.6	0.2	Not amplified	Not applicable
CCUG 20309	A^−^ B^+^	y = −3.43×+41.2	1.2	y = −3.66×+45.0	0.6	Not amplified	Not applicable
ATCC 700057	A^−^ B^−^	y = −3.52×+42.1	2.1	Not amplified	Not applicable	Not amplified	Not applicable
CCUG 37780	A^−^ B^−^	y = −3.47×+40.3	0.3	Not amplified	Not applicable	Not amplified	Not applicable
CCUG 37785	A^−^ B^−^	y = −3.44×+40.7	0.7	Not amplified	Not applicable	Not amplified	Not applicable

a Each analytical curve of different *C. difficile* strains was generated with serial dilutions ranging from 10 to 10^5^ cells per reaction. X-axis is bacterial cells applied to the reaction (log_10_ cells/reaction) and Y-axis is the *C_T_* values obtained.

b Differences in *C_T_* values compared with that of the type strain (DSM 1296^T^) are indicated.

### Comparison of analytical curve of *C. difficile*-spiked stool with that of *C. difficile* pure culture

A stool specimen lacking any amplification by qPCR using CD16SrRNA-F/R/P was selected, as this response suggested that the specimen lacked indigenous populations of *C. difficile*. The *C. difficile*-negative stool was diluted with Dulbecco’s PBS (–) to make a 10% (w/v) stool homogenate (100 mg stool/mL). The number of cells in a pure culture of *C. difficile* DSM 1296^T^ in 1% Glc-mGAM broth was counted by using DAPI staining, and serial dilutions of the pure culture ranging from 10^4^ to 10^9^ cells/mL were prepared. Twenty microliters of the serial dilutions (containing *C. difficile* cells ranging from 2×10^2^ to 2×10^7^) were spiked into the 2 mL of the *C. difficile*-negative stool homogenates (containing 200 mg of stool) to obtain stool specimens with *C. difficile* at final concentrations ranging from 10^3^ to 10^8^ cells/g of stool. DNA extracted from the stool specimens was applied to qPCR, and the obtained *C_T_* values were used to generate an analytical curve of the *C. difficile*-spiked stool. DNA extracted from 2×10^7^ cells of pure cultured *C. difficile* and its serial dilutions were applied to qPCR to generate a standard analytical curve of the *C. difficile* pure culture. These two analytical curves were compared to evaluate the lower detection limit and detection accuracy of this qPCR method.

### Determination of bacterial count by qPCR

Whole *C. difficile*, TcdA-producing strains, and TcdB-producing strains were enumerated by qPCR with 16SrRNA-F/R/P, tcdA-F/R/P, and tcdB-F/R/P, as follows. *C. difficile* DSM 1296^T^ A^+^B^+^ strain was selected as a standard strain for generating standard analytical curves for all three target *C. difficile* groups. Five microliters of 10-fold serial dilutions of DNA extracted from the pure culture of the *C. difficile* strain were applied to PCR to obtain a standard analytical curve ranging from 10^1^ to 10^5^ cells/5-µL reaction. Five microliters of the DNA solution extracted from 200 mg of stool and its 2- and 4-fold dilutions were applied to PCR as a template containing the corresponding DNA from 10, 5, or 2.5 mg of stool. The consequent *C_T_* values of the stool specimens were applied to the standard analytical curve, and the corresponding bacterial counts (cells/g of stool) were calculated as qPCR counts.

### Determination of toxigenic types of *C. difficile* predominating in individual stool specimens

By comparing the three qPCR counts targeting the 16S rRNA gene for whole *C. difficile* (A^+^B^+^, A^−^B^+^, and A^−^B^−^ types), *tcdB* for TcdB-producing strains (A^+^B^+^ and A^−^B^+^ types), and *tcdA* for TcdA-producing strains (A^+^B^+^ type), the toxigenic types of *C. difficile* predominating in each stool specimen were determined as follows. When the differences among the three qPCR counts were within 0.3 log_10_ cells/g of stool (a 2-fold difference in real values), the toxigenic type of the dominant *C. difficile* strain was identified as A^+^B^+^. When the difference between the qPCR counts of the 16S rRNA gene and *tcdB* was within 0.3 log_10_ cells/g of stool and these counts were higher than that of *tcdA* by at least 0.3 log_10_ cells/g of stool, the toxigenic type was identified as A^−^B^+^. When the qPCR count of the 16S rRNA gene was higher than those of *tcdA* and *tcdB* by at least 0.3 log_10_ cells/g of stool, the toxigenic type was identified as A^−^B^−^.

### Enzyme immunoassay (EIA)

*C. difficile* toxins in stools were detected with a commercial EIA kit, the Xpect *Clostridium Difficile* Toxin A/B Test (Remel Inc., Lenexa, KS), in accordance with the manufacturer’s instructions.

### *C. difficile* selective culture (CDSC)

In accordance with the method described by Wren [14], *C. difficile* was isolated by stool culture with cefoxitin cycloserine egg yolk (CCEY) agar. CCEY agar (BioConnections Ltd., Knypersley United Kingdom) supplemented with 40 mL of egg yolk emulsion (BioConnections), two vials of cefoxitin-cycloserin (BioConnections), and 10 mL of lysed horse blood per liter was prepared in-house and stored at 4°C for a maximum of 1 week before use. The 10% (w/v) stool homogenate was mixed with an equal volume of absolute ethanol and left at room temperature for 30 to 60 min. In an anaerobic glovebox (Coy Laboratory Products Inc., Grass Lake, MI), 100 µL of the alcohol-treated stool or its 10-fold dilution was inoculated onto the CCEY plates and cultured under anaerobic conditions at 37°C for 48 to 72 h. Suspected colonies of *C. difficile* were selected on the basis of their morphological characteristics and subjected to rapid identification by real-time PCR with the CD16SrRNA-F/R/P set. Obtained *C. difficile* isolates were examined by PCR for identification of toxigenic types and by cell cytotoxicity neutralization assay (CCNA) for the detection of TcdB.

### Identification of toxigenic type of *C. difficile* isolates by PCR

Toxigenic types of *C. difficile* isolates were identified by comparing the size of PCR amplicons for the respective toxin genes, in accordance with the method described by Kato *et al.* [15]. An NK11/NK9 primer set for *tcdA* and an NK104/NK105 primer set for *tcdB* were used for amplification.

### TcdB detection of *C. difficile* isolates by CCNA

The cytopathic effect of the isolates was examined with a *C. difficile* Toxin/Antitoxin Kit (Techlab, Blacksburg, VA). *C. difficile* isolates were subcultured in BHI broth anaerobically at 37°C for 5 days. The suspension was then centrifuged at 16,000*×g* for 5 min and the supernatant filtered with a 0.45-µm membrane filter. The filtrate was added to precultured Vero-B4 cells with or without *C. difficile* antitoxin. After incubation of the culture at 37°C for 24 or 48 h, the presence or absence of TcdB was determined by judging the cytopathic effects. The following criteria were employed: when at least 90% of the cells were rounded in the absence of antitoxin and the cytopathic effect was neutralized by the antitoxin, the culture was regarded as TcdB positive; when no cytopathic effect was observed in the cells both with and without antitoxin, the culture was regarded as TcdB negative.

## Results

### Selectivity of qPCR using primers-probe sets targeting the 16S rRNA, *tcdA*, and *tcdB* genes

For selective detection of whole *C. difficile* (A^+^B^+^, A^−^B^+^, and A^−^B^−^ types), TcdB-producing strains (A^+^B^+^ and A^−^B^+^ types), and TcdA-producing strains (A^+^B^+^ type), respective primers-probe sets targeting 16S rRNA, *tcdB*, and *tcdA* genes were newly designed (Table 1). The selectivity of qPCR using the three designed primers-probe sets was assessed by using DNA extracted from each pure culture (Table 2). qPCR using each primers-probe set detected the respective target strains selectively, without any amplification of non-targeted strains. To evaluate the accuracy of quantitative detection among the target *C. difficile* strains, each analytical curve was compared (Table 3). The analytical curves of the 10 *C. difficile* strains (five A^+^B^+^ strains, two A^−^B^+^ strains, and three A^−^B^−^ strains) with 16SrRNA-F/R/P had almost equal slopes. Similarly, for the seven TcdB-producing strains (five A^+^B^+^ and two A^−^B^+^ strains) with tcdB-F/R/P and the five TcdA-producing strains (five A^+^B^+^ strains) with tcdA-F/R/P, the slopes of the analytical curves were equivalent. The differences in the intercepts of the analytical curves with 16SrRNA-F/R/P between the type strain and the others ranged from –0.9 to +2.1, suggesting that each target strain could be enumerated within approximately a 4-fold difference. The difference in the intercepts ranged from –1.0 to +0.8 in the case of analytical curves with tcdB-F/R/P and from –0.3 to +1.5 in the case of those with tcdA-F/R/P, thus providing further evidence of the accurate quantification of toxigenic strains. These results indicated that the newly developed TaqMan-based qPCR method was capable of detecting the target *C. difficile* strains selectively and with high accuracy.

### Lower detection limit and detection accuracy of qPCR

**Figure 1:**
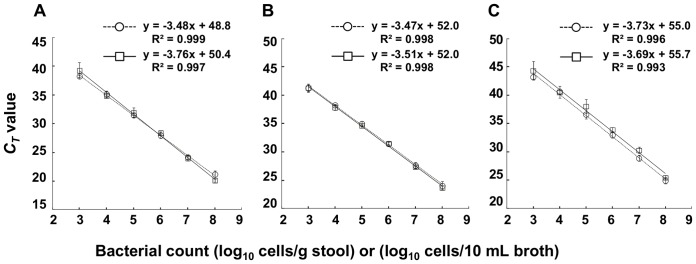
qPCR quantification of *C. difficile* DSM 1296^T^ (A^+^B^+^ strain) spiked into a human stool. Stool samples taken from a healthy adult and supplemented with serial dilutions of *C. difficile* DSM 1296^T^ (A^+^B^+^ strain) at final concentrations ranging from 10^3^ to 10^8^ cells/g of stool were examined by qPCR using CD16SrRNA-F/R/P (A), tcdA-F/R/P (B), or tcdB-F/R/P (C). Cell counts of the spiked *C. difficile* were determined by DAPI staining. The obtained analytical curve of the *C. difficile*-spiked stool (□) was compared with the standard analytical curve of the *C. difficile* pure culture (○).

The lower detection limit and detection accuracy of qPCR for *C. difficile* in stools were evaluated by analyzing stool samples spiked with *C. difficile* vegetative cells at a final concentration of 10^3^ to 10^8^ cells/g of stool. The analytical curve of the *C. difficile*-spiked stool was compared with the standard analytical curve of the *C. difficile* pure culture (Figure 1A–C). In the case of all the three primers-probe sets, the obtained analytical curves were nearly equivalent over the range of 10^3^ to 10^8^ cells, confirming that qPCR with the standard analytical curve of the pure culture allowed accurate detection of *C. difficile* in stools. These results also indicated that our qPCR method enabled quantitative detection of *C. difficile* in stools with a lower detection limit of 10^3^ cells/g of stool.

### Comparison of results of toxigenic *C. difficile* detection by qPCR and CDSC

To verify the validity of the new TaqMan-based qPCR method, we compared the results of *C. difficile* detection in stool specimens by qPCR with those by CDSC as a reference method. Stool specimens were collected from 82 elderly people in four different nursing homes once every 3 months for 6 months, i.e. a total of three times (S_1_, S_2_, and S_3_ sampling). A total of 235 specimens were examined for *C. difficile* by qPCR and CDSC (Table 4). *C. difficile* was detected in 12 of the 235 specimens by qPCR, whereas this organism was isolated by CDSC from 9 specimens; there were thus three discrepancies between the methods. *C. difficile* toxins were not detected in any of the 235 specimens by EIA (data not shown).

**Table 4:** Comparison of detection results of *C. difficile* between qPCR and *C. difficile* selective culture (CDSC).

qPCR result^a^	No. of specimens with CDSC result^b^		Total
	*C. difficile* positive	*C. difficile* negative	
***C. difficile*** ** positive**	9	3	12
***C. difficile*** ** negative**	0	223	223
**Total**	9	226	235

a “*C. difficile* positive/negative” was defined by presence/absence of qPCR amplification with the 16S rRNA primers-probe set.

b “*C. difficile* positive/negative” was defined by presence/absence of *C. difficile* isolation by means of stool culture.

Details of the test results from the 12 *C. difficile*-positive specimens from eight subjects are shown in Table 5. Whole *C. difficile* counts (16S rRNA target) ranged from 10^3^ to 10^5^ cells level/g of stool, with the exception of one specimen, #02010-S_3_, which had a much higher count. The mean qPCR counts of whole *C. difficile*, TcdB-producing strains, and TcdA-producing strains were 4.5±1.3, 4.6±0.4, and 4.5±0.6 log_10_ cells/g of stool, respectively. On the basis of the qPCR counts for the three genes within each specimen, the toxigenic type of the predominating *C. difficile* was identified as A^+^B^+^ in six specimens (#02007-S_2_, #03008-S_1_, #03008-S_2_, #03008-S_3_, #03024-S_2_, #04003-S_3_) and as A^−^B^−^ type in the other six specimens (#02010-S_3_, #02011-S_3_, #04011-S_2_, #04026-S_1_, #04026-S_2_, #04026-S_3_). The PCR analysis on CDSC revealed that the isolates from six specimens were of A^+^B^+^ type and those from the remaining three specimens were A^−^B^−^ type. The A^+^B^+^ isolates from the six specimens were confirmed by cell cytotoxicity assay to be capable of producing TcdB, although the toxin was not detected in any of the stool specimens by EIA. The toxin-production profiles of these isolates, as determined by CDSC analysis, were consistent with those determined by qPCR. In terms of the results of toxigenic *C. difficile* detection, both qPCR and CDSC gave the same six positive specimens, indicating that qPCR was as efficient in detecting toxigenic *C. difficile* in stools as CDSC.

**Table 5:** Comparison of detection results of toxigenic *C. difficile* or toxins by qPCR, *C. difficile* selective culture (CDSC), and enzyme immunoassay (EIA).

Subject ID	Specimen	qPCR				CDSC			EIA
		Counts (log_10_ cells/g of stool)			Toxigenic type^a^	*C. difficile* isolation	Isolates test		
		16S rRNA	*tcdA*	*tcdB*			Toxigenic type^b^^,^ c	Cell cytotoxicity^c^	
02007	S_2_	4.4	4.5	4.7	A^+^ B^+^	Yes	A^+^ B^+^	Pos	Neg
02010	S_3_	8.0	<3.0	<3.0	A^−^ B^−^	Yes	A^−^ B^−^	Neg	Neg
02011	S_3_	3.3	<3.0	<3.0	A^−^ B^−^	No	na	na	Neg
03008	S_1_	5.1	5.1	5.1	A^+^ B^+^	Yes	A^+^ B^+^	Pos	Neg
	S_2_	4.4	4.4	4.6	A^+^ B^+^	Yes	A^+^ B^+^	Pos	Neg
	S_3_	4.5	4.6	4.7	A^+^ B^+^	Yes	A^+^ B^+^	Pos	Neg
03024	S_2_	3.5	3.5	3.8	A^+^ B^+^	Yes	A^+^ B^+^	Pos	Neg
04003	S_3_	5.0	5.0	4.9	A^+^ B^+^	Yes	A^+^ B^+^	Pos	Neg
04011	S_2_	4.3	<3.0	<3.0	A^−^ B^−^	No	na	na	Neg
04026	S_1_	4.7	<3.0	<3.0	A^−^ B^−^	Yes	A^−^ B^−^	Neg	Neg
	S_2_	3.5	<3.0	<3.0	A^−^ B^−^	No	na	na	Neg
	S_3_	3.0	<3.0	<3.0	A^−^ B^−^	Yes	A^−^ B^−^	Neg	Neg
Mean		4.5	4.5	4.6					
SD		1.3	0.6	0.4					

a Toxigenic types were identified on the basis of qPCR counts for the three genes, according to the criteria described in Materials and Methods.

b The toxigenic type of isolates was determined on the basis of PCR amplification of *tcdA* and *tcdB* by using the method of Kato *et al* [15].

c na, not applicable.

### *C. difficile* carriage in the elderly in nursing homes

**Figure 2:**
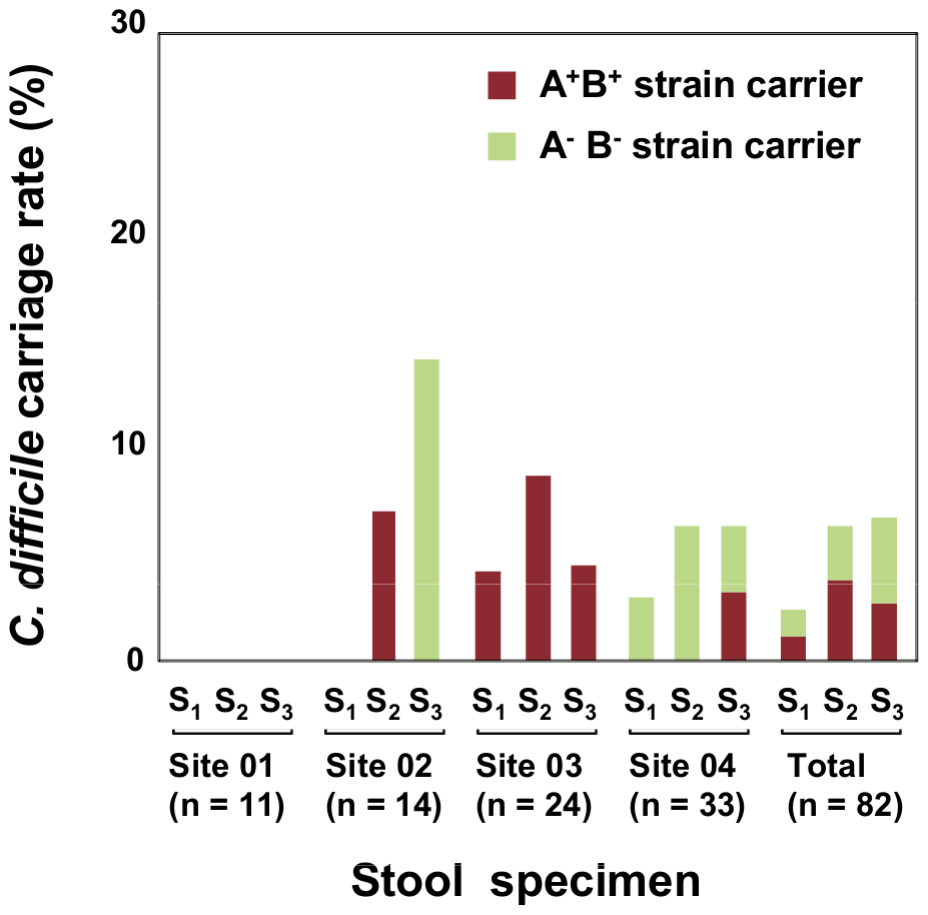
*C. difficile* carriage rates in four nursing home populations. On the basis of the qPCR counts for three genes (16S rRNA, *tcdA*, and *tcdB*), the toxigenic types (A^+^B^+^, A^−^B^+^, or A^−^B^−^) of *C. difficile* predominating in individual specimens were identified. The rates of carriage of each toxigenic type of *C. difficile* at three stool samplings (S1, S2, and S3) were calculated with respect to each nursing home (n = 11, 14, 24, and 33, respectively) and the total population (n = 82).

We obtained detection rates and qPCR counts of whole *C. difficile*, TcdB-producing strains, and TcdA-producing strains in elderly residents of different nursing homes at three samplings (S_1_∶82 specimens; S_2_∶79 specimens; and S_3_∶74 specimens) (See Table S2). Detection rates of whole *C. difficile* in S_1_, S_2_, and S_3_ specimens from the four nursing homes were 2/82 (2.4%), 5/79 (6.3%), and 5/74 (6.8%), respectively. The detection rates of TcdA-producing strains were 1/82 (1.2%), 3/79 (3.8%), and 2/72 (2.7%), respectively, and those of TcdB-producing strains were exactly the same. On the basis of the qPCR counts, we determined the toxigenic types of the predominating *C. difficile* in each specimen; the rates of carriage of the respective types by the 82 subjects are shown in Figure 2. Whereas site 01 had no *C. difficile* carrier throughout the 6-month period, the other sites had some carriers of either the A^+^B^+^ strain or the A^−^B^−^ strain, or both. The overall carriage rate of *C. difficile* in the 82 subjects fluctuated below 10% during the 6-month test period, except in one case (S_3_ at site 02).

As a result of review of medical records, 19 (23%) of 82 subjects received at least one course of antibiotic treatment in the study period of 6 months. *C. difficile* was detected in only one of the 19 subjects (#02010). The number of subjects who had hospitalization during the study period was six (7.3%), only one of which, the same #02010, was *C. difficile*-positive. The subject underwent several antibiotic treatments and short-term hospitalizations between S_2_ and S_3_ sampling.

## Discussion

We designed new oligonucleotide sets targeting the 16S rRNA, *tcdB*, and *tcdA* genes for the quantification of whole *C. difficile* (A^+^B^+^, A^−^B^+^, and A^−^B^−^ types), TcdB-producing strains (A^+^B^+^ and A^−^B^+^ types), and TcdA-producing strains (A^+^B^+^ type), respectively. Most clinically isolated TcdA-negative, TcdB-positive strains (A^−^B^+^ type) possess *tcdA* that has a 1.8-kbp deletion in 8.0 kbp of the intact full length sequence [16]–[19], and therefore does not produce functional TcdA. We designed *tcdA*-specific primers and a probe against this deleted region, and confirmed that our PCR correctly identified intact *tcdA* genes and did not detect *tcdA* genes that were rendered non-functional due to the deletion (Table 2). Moreover, these newly developed primers-probe sets were designed against the highly conserved region of each gene and consequently included no mismatches with their target sequences. Indeed, qPCR was capable of detecting all the strains in the target group with almost equal reactivity (Table 3). Up to the present, many sets of primers and probes targeting toxin genes have been reported [6], [20]–[22]. However, *in silico* comparison of those oligonucleotides with their target gene sequences currently available in the database revealed that most of them included at least a few mismatches against some of their targets (data not shown); this may result in inaccurate quantification due to strain-dependent differences in reactivity. It can be expected that our qPCR method overcame this potential defect.

The lower detection limit of *C. difficile* in stools remained to be solved. We previously reported that reverse transcription (RT)-qPCR targeting the rRNA molecule achieved sensitive detection of *C. difficile* with a lower detection limit of 10^2.4^ cells/g of stool [23]. However, this application was limited to *C. difficile* species because such a low detection limit was accomplished only by targeting rRNA molecules present in high copy numbers (10^3^ to 10^4^ molecules per single cell) [24], [25]. Here, we developed a detection system for toxigenic strains that was based on qPCR targeting the toxin genes. To lower the limit of detection by qPCR, we subjected a highly concentrated stool DNA fraction to PCR in the presence of an agent that neutralized PCR amplification inhibitors. In general, extraction of DNA from larger amounts of stool and dissolution in smaller amounts of buffer can yield highly concentrated DNA, but the product will contain increased amounts of stool-derived PCR inhibitors. In our study, a stool DNA fraction that was prepared by using a commercial DNA extraction kit (FastDNA SPIN Kit for Feces) and contained DNA from as much as 10 mg of stool was subjected to qPCR without dilution because the commercial PCR buffer (Ampdirect Plus) neutralized the effect of the PCR inhibitors. This combination successfully lowered the detection limit to 10^3^ cells/g of stool (Figure 1). We confirmed that an application of the extracted stool DNA solution to PCR without Ampdirect plus gave no amplification (data not shown). The qPCR counts of *C. difficile* in most subjects fell into a low range (e.g. 10^3^ or 10^4^ cells/g of stool; Table 5), indicating that the lower detection limit of 10^3^ cells/g of stool is important in the in-depth investigation of *C. difficile* present in stools of asymptomatic subjects.

In our examinations, the consistent result of *C. difficile* detection was obtained in 232 out of 235 specimens between qPCR and CDSC (Table 4), supporting the validity of our TaqMan PCR as a sensitive method to detect *C. difficile* in stools. The other three specimens were qPCR positive but CDSC negative. One possible reason of this discrepancy is the difference of *C. difficile* forms targeted by the methods. *C. difficile* has typically two forms; one is the active and infectious form, vegetative cells, and the other is the inactive form, spores. In our CDSC examination, *C. difficile* spores were exclusively detected since alcohol treatment killed vegetative cells. In contrast, we confirmed that our qPCR method mainly detected vegetative cells because the DNA extraction efficiency from spores was approximately 1,000-times as low as that from vegetative cells (data not shown). The discrepant result between qPCR and CDSC in the three specimens may therefore reflect that *C. difficile* present in the stools was composed of the majority of vegetative cells and the minority of spores, and that a smaller amount of spores were undetectable by CDSC. In our study, CDSC was performed for the purpose of detecting only spores because it was thought to be difficult to meet all the requirements for the detection of vegetative cells by culture, such as collection of fresh stools with less exposure to aerobic environment, more rapid transportation of stool from study sites to our laboratory, and immediate examination. An application of a cultural procedure for the detection of vegetative cells as well as spores could have provided further information. In addition, modification of DNA extraction method to improve the extraction efficiency from spores would make it possible to compare detection results of the same target, both vegetative cells and spores, between qPCR and culture methods. Another possible reason of the discrepancy is an issue of living and dead cells. In general, while a culture method detects only living cells, conventional qPCR detects both living and dead cells, which could result in the higher detection rate of *C. difficile* by qPCR. Recently, selective detection of living cells by PCR in combination with cell membrane-impermeable dyes, which modify DNA of dead cells and inhibit PCR amplification, has been reported [26], [27]. We consider it necessary to apply such living cell-specific detection system to our qPCR method for the detection of authentic living bacterial cells in stools. Other reasons such as false-positive results of qPCR may also lead to discrepancy. Although the primers-probe set was carefully checked to specifically detect *C. difficile*, it remains a possibility of non-specific reaction with other unknown bacteria. Despite these differences in the methodological character of qPCR and CDSC, the highly comparable results of *C. difficile* detection were obtained (Table 4), suggesting the effectiveness of our qPCR targeting vegetative cells as the detection method of *C. difficile* in stools.

The *C. difficile* carriage rate in long-term care facilities (LTCFs) is considered higher than that in community-dwelling adults but lower than that in hospital inpatients [28]. In our previous study of 83 LTCFs residents in Japan [23], we revealed that the carriage rate of *C. difficile* was as high as 43%. Riggs *et al.* [5] reported a 51% carriage rate of toxigenic *C. difficile* in 68 inpatients of LTCFs in Ireland, as determined by using a culture method. However, in our present study of 82 nursing home residents in France, the carriage rates of *C. difficile* and toxigenic *C. difficile*, as determined by TaqMan-based qPCR over 6 months, ranged from 2.4% to 6.8% and 1.2% to 3.8%, respectively-much lower than those reported in the studies above. Nevertheless, the *C. difficile* carriage rates reported in other culture-based examinations are comparable to our current results [3], [4], [29], [30]. For example, Walker *et al.* [30] reported that the carriage rates of whole *C. difficile* and toxigenic *C. difficile* in 225 LTCF residents in the United States were 7.1% and 4.0%, respectively. In a later study by Arvand *et al*. [29], these carriage rates in 240 elderly nursing home residents in Germany were 4.6% and 4.2%, respectively. LTCFs include various facilities, such as nursing homes, rehabilitation facilities, inpatient behavioral health facilities, and long-term chronic care hospitals. Because environmental contamination with *C. difficile* occurs commonly in hospitals [31], [32], subjects in facilities close to hospital environments are likely to have more chances to acquire the organism. Certainly, the facilities in our study, which had relatively low *C. difficil*e carriage rates, were nursing homes, whereas those in our previous study, which had higher carriage rates, were chronic care facilities. Thus, difference in the types of LTCFs may explain these variations in *C. difficile* carriage rates.

Exposure to antibiotics and frequent or prolonged hospitalization are the major risk factors for acquisition or colonization of *C. difficile* [31], [33], [34]. In our study, subject #02010, who had several antibiotic treatments and hospital stays between S_2_ and S_3_, acquired *C. difficile* at S_3_, and the qPCR count was over 10^8^ cells/g of stool– much higher than in the other subjects (Table 5). It is likely that overgrowth of *C. difficile* newly acquired from the hospital environment was observed by the TaqMan-based qPCR, although this was not a case of CDI because the strain was non-toxigenic. All eight of our *C. difficile*-positive subjects, including the four toxigenic *C. difficile* carriers, had no abdominal symptoms. The mean qPCR count of *C. difficile* in these asymptomatic carriers was 4.5 log_10_ cells/g of stool (Table 5). Naaber *et al.* [35] examined stools from patients with antibiotic-associated diarrhea by using *C. difficile* species-level qPCR. They reported increased numbers of *C. difficile*, ranging from 5.6 to 11.2 log_10_ cells/g of stool. They also revealed that the mean qPCR count in *C. difficile-*toxin-positive stools was higher than that in toxin-negative stools (9.3 vs. 6.3 log_10_ cells/g of stool). Riggs *et al.* [5] also reported that the mean *C. difficile* count in 18 patients with CDI was higher than that in 20 asymptomatic carriers (5.6 vs. 3.6 CFU/g of stool). Thus, the bacterial number in stools may be useful for predicting the status of *C. difficile* carriers (i.e. symptomatic or asymptomatic) and the severity of symptoms. Our TaqMan-based qPCR method would be appropriate for such assessments because it enables accurate monitoring of *C. difficile* counts with an appropriate lower detection limit. We believe that use of this method could provide valuable information for the control of CDI.

In conclusion, we developed a sensitive and selective detection system for *C. difficile* in human stools that uses TaqMan-based qPCR. Application of qPCR to the examination of stools from nursing home residents revealed in detail the prevalence of *C. difficile*, including toxigenic strains, indicating that this method can be an effective tool for both clinical diagnosis and epidemiological investigation.

## Supporting Information

Table S1 **Accession numbers of nucleotide sequences used for the design of primers and probes.**
(DOCX)

Table S2 **qPCR result of** ***C. difficile*** **detection in four nursing home populations over 6months.**
(DOCX)
