# Supplementary material for: Development of TaqMan-Based Quantitative PCR for Sensitive and Selective Detection of Toxigenic Clostridium difficile in Human Stools
Source: PLoS One. 2014 Oct 31;9(10):e111684. doi: 10.1371/journal.pone.0111684 (PMC4216139; doi:10.1371/journal.pone.0111684)
Supplement: Table S1 — Accession numbers of nucleotide sequences used for the design of primers and probes. (DOCX) [file pone.0111684.s001.docx]

**Table S1. Accession numbers of nucleotide sequences used for the design of primers and probes.**

| **Gene** | **No. of sequence** | **Database and accession number** |
| --- | --- | --- |
| 16S rRNA gene | 15 | Ribosomal database project; S000006023, S000130063, S000260455, S000428428, S000428429, S001611434, S001611472, S002038933, S002225098, S002287333, S002448641, S002448642, S002949118, S002949119, S002949120 |
| *tcdA* | 14 | GenBank; M30307, NC_009089, NC_013316, NC_013315, AJ011301, NZ_ADVM01000023, NZ_ABHF02000018, NZ_ABHE02000016, NZ_ABFD02000006, NZ_ABHD02000008, NZ_ABHG02000011, NZ_ABKK02000013, NZ_AAML04000007, NZ_ABKL02000008 |
| *tcdB* | 17 | GenBank; AJ011301, NC_009089, NC_013316, NC_013315, FN665654, AF217292, X53138, NZ_ABHF02000018, NZ_ADVM01000023, NZ_ADNX01000011, NZ_ABHE02000016, NZ_ABHD02000008, NZ_ABFD02000006, NZ_ABKL02000008, NZ_ABKK02000013, NZ_ABHG02000011, NZ_AAML04000007 |
