# Supplementary material for: Development of TaqMan-Based Quantitative PCR for Sensitive and Selective Detection of Toxigenic Clostridium difficile in Human Stools
Source: PLoS One. 2014 Oct 31;9(10):e111684. doi: 10.1371/journal.pone.0111684 (PMC4216139; doi:10.1371/journal.pone.0111684)
Supplement: Table S2 — qPCR result of C. difficile detection in four nursing home populations over 6months. (DOCX) [file pone.0111684.s002.docx]

**Table S2. qPCR result of *C. difficile* detection in four nursing home populations over 6 months.**

|  |  | **Detection result of *C. difficile* groups by qPCR with the following oligonucleotide sets:** | | | | | |
| --- | --- | --- | --- | --- | --- | --- | --- |
|  |  | **CD16SrRNA-F/R/P** | | **tcdA-F/R/P** | | **tcdB-F/R/P** | |
| **Stool specimen^a^** | | **Detection rate^b^** | **Count^c, d^** | **Detection rate^b^** | **Count^c, d^** | **Detection rate^b^** | **Count^c, d^** |
| Site 01 | S_1_ | 0/11 (0%) | na | 0/11 (0%) | na | 0/11 (0%) | na |
|  | S_2_ | 0/10 (0%) | na | 0/10 (0%) | na | 0/10 (0%) | na |
|  | S_3_ | 0/7 (0%) | na | 0/7 (0%) | na | 0/7 (0%) | na |
| Site 02 | S_1_ | 0/14 (0%) | na | 0/14 (0%) | na | 0/14 (0%) | na |
|  | S_2_ | 1/14 (7.1%) | 4.4 | 1/14 (7.1%) | 4.5 | 1/14 (7.1%) | 4.7 |
|  | S_3_ | 2/14 (14.3%) | 5.7 | 0/14 (0%) | na | 0/14 (0%) | na |
| Site 03 | S_1_ | 1/24 (4.2%) | 5.1 | 1/24 (4.2%) | 5.1 | 1/24 (4.2%) | 5.1 |
|  | S_2_ | 2/23 (8.7%) | 4.0 | 2/23 (8.7%) | 3.9 | 2/23 (8.7%) | 4.2 |
|  | S_3_ | 1/22 (4.5%) | 4.5 | 1/22 (4.5%) | 4.6 | 1/22 (4.5%) | 4.7 |
| Site 04 | S_1_ | 1/33 (3.0%) | 4.7 | 0/33 (0%) | na | 0/33 (0%) | na |
|  | S_2_ | 2/32 (6.3%) | 3.9 | 0/32 (0%) | na | 0/32 (0%) | na |
|  | S_3_ | 2/31 (6.5%) | 4.0 | 1/31 (3.2%) | 5.0 | 1/31 (3.2%) | 4.9 |
| Total | S_1_ | 2/82 (2.4%) | 4.9 | 1/82 (1.2%) | 5.1 | 1/82 (1.2%) | 5.1 |
|  | S_2_ | 5/79 (6.3%) | 4.0 ± 0.5 | 3/79 (3.8%) | 4.1 ± 0.6 | 3/79 (3.8%) | 4.4 ± 0.5 |
|  | S_3_ | 5/74 (6.8%) | 4.8 ± 2.0 | 2/74 (2.7%) | 4.8 | 2/74 (2.7%) | 4.8 |

^a^ Stool specimens collected once every 3 months (in total three times over 6 months) were examined by qPCR.

^b^ Detection of each *C. difficil*e group was defined by a qPCR count of at least 3.0 log_10_ cells/gram of stool.

^c^ Mean ± SD (log_10_ cells/g of stool)

^d^ na, not applicable
